# Shallow subtidal marine benthic communities of Nachvak Fjord, Nunatsiavut, Labrador: A glimpse into species composition and drivers of their distribution

**DOI:** 10.1371/journal.pone.0293702

**Published:** 2023-11-09

**Authors:** Alan M. Friedlander, Enric Ballesteros, Alyssa M. Adler, Whitney Goodell, Ryan Jenkinson, Jennie A. Knopp, Christopher D. H. Thompson, Molly Timmers, Cameron A. J. Walsh, Enric Sala

**Affiliations:** 1 Pristine Seas, National Geographic Society, Washington, DC, United States of America; 2 Hawaiʿi Institute of Marine Biology, University of Hawaiʿi, Kāneʻohe, Hawaiʿi, United States of America; 3 Centre d’Estudis Avançats de Blanes-CSIC, Blanes, Girona, Spain; 4 Division of Marine Science and Conservation, Nicholas School of the Environment, Duke University, Beaufort, North Carolina, United States of America; 5 Hawaiʿi Division of Aquatic Resources, Department of Land and Natural Resources, Honolulu, Hawaiʿi, United States of America; 6 Oceans North, Ottawa, Canada; 7 Marine Futures Lab, School of Biological Sciences, University of Western Australia, Crawley, West Australia, Australia; The University of Auckland - City Campus: University of Auckland, NEW ZEALAND

## Abstract

Marine fjords along the northern Labrador coast of Arctic Canada are influenced by freshwater, nutrients, and sediment inputs from ice fields and rivers. These ecosystems, further shaped by both Atlantic and Arctic water masses, are important habitats for fishes, marine mammals, seabirds, and marine invertebrates and are vital to the Labrador Inuit who have long depended on these areas for sustenance. Despite their ecological and socio-cultural importance, these marine ecosystems remain largely understudied. Here we conducted the first quantitative underwater scuba surveys, down to 12 m, of the nearshore marine ecology of Nachvak Fjord, which is surrounded by Torngat Mountains National Park located in Nunatsiavut, the Indigenous lands claim region of northeastern Canada. Our goal was to provide the Nunatsiavut Government with a baseline of the composition and environmental influences on the subtidal community in this isolated region as they work towards the creation of an Indigenous-led National Marine Conservation Area that includes Nachvak Fjord. We identified four major benthic habitat types: (1) boulders (2) rocks with sediment, (3) sediment with rocks, and (4) unconsolidated sediments, including sand, gravel, and cobble. Biogenic cover (e.g., kelp, coralline algae, and sediment) explained much of the variability in megabenthic invertebrate community structure. The kelp species *Alaria esculenta*, *Saccharina latissima*, and *Laminaria solidungula* dominated the boulder habitat outside of the fjord covering 35%, 13%, and 11% of the sea floor, respectively. In contrast, the middle and inner portions of the fjord were devoid of kelp and dominated by encrusting coralline algae. More diverse megabenthic invertebrate assemblages were detected within the fjord compared to the periphery. Fish assemblages were depauperate overall with the shorthorn sculpin, *Myoxocephalus scorpius*, and the Greenland cod, *Gadus ogac*, dominating total fish biomass contributing 64% and 30%, respectively. Understanding the composition and environmental influences within this fjord ecosystem not only contributes towards the protection of this ecological and culturally important region but serves as a baseline in a rapidly changing climatic region.

## Introduction

In Arctic marine ecosystems, the seasonal and geographical variation of sea ice directly influences the physical, chemical, and biological characteristics of pelagic and coastal environments [1–3]. Sea ice reduces sea surface temperatures, restricts light availability, diminishes water flow, and alters salinity for long periods of the year. Despite these seemingly harsh environmental limitations, the Arctic Ocean is brimming with life and has remained in a relatively healthy ecological state owing to the low human population density and limited anthropogenic impacts in the region [4]. However, both pelagic and benthic habitats in the Arctic are highly vulnerable to ocean warming as most of their differential features depend on sea ice and its duration for ecosystem function [3, 5]. As a result, this once relatively intact and untouched marine ecosystem has become one of the most vulnerable on the planet [6–8].

Coastal marine ecosystems are particularly sensitive to warming [9–11] and Arctic shores, which harbor unique coastal marine communities, make up a third of the world’s coastline [12]. The Canadian Arctic alone hosts 162,000 km of Arctic shoreline (10% of the world’s coastline) [13, 14] and while several studies have investigated the species living on Canada’s Arctic shorelines and offshore seafloor habitats, little effort has been devoted to the nearshore shallow marine communities (< 30 m) despite their vulnerability under ocean warming [15–17]. There is a near-complete absence of published information on the habitats and assemblages on nearshore Canadian Arctic waters. This scarcity of literature is in part due to Canada’s vast, inaccessible, and irregular shoreline, the exception being Hudson Bay [4].

Studies of the fjord lined coasts of Nunatsiavut, the Indigenous lands claim region along the Labrador coast of northeastern Canada, are also limited in scope and geography. There are only a handful of peer-reviewed published studies to date for the region, which provide some information on kelp distribution [14, 18], fjord geomorphology [19], seasonal and environmental phytoplankton [20], and sediment derived dinoflagellates [21]. This limitation is in part due to the remoteness and vast number of marine fjords that line the coastline. These inaccessible and irregular shorelines are influenced by both Atlantic and Arctic water masses and receive freshwater, nutrients, and sediments from rivers and streams [22]. They form important habitats due to their high bathymetric relief, geomorphic complexity, unique circulation patterns, and natural gradients in temperature, salinity, oxygenation, and other water column properties [23]. They are important feeding grounds for fishes, marine mammals, seabirds, and marine invertebrates. Furthermore, these marine fjord ecosystems have contributed to the well-being, subsistence, and food security for Labrador Inuit since time immemorial [24, 25]. Despite their ecological and socio-cultural importance, the fjords of Nunatsiavut remain largely understudied [22, 26].

In 2005, Torngat Mountains National Park Reserve (Tongait KakKasuangita SilakKijapvinga; designated Torngat Mountains National Park in 2008) was officially established with the intent of protecting the unique natural history and culture of this region [27, 28]. The park takes its name from the Inuktitut word *Tongait*, meaning place of spirits [29], and surrounds the fjords in the northern reaches of the region. Currently the land surrounding most of the fjords within the Nunatsiavut land claims region is under protection through the creation of the Torngat Mountains National Park. The Nunatsiavut Government is working in collaboration with Parks Canada on a feasibility assessment to extend existing terrestrial protections into the marine regions, extending off the coast as a National Marine Conservation Area.

The aims of this study were to: 1) provide a baseline record of the community composition and environmental influences on the subtidal marine life of Nachvak Fjord, one of many remote fjords along the Nunatsiavut coastline and 2) fill data gaps and provide data necessary towards the protection of these marine waters. Owing to the changing climate in this region [26], understanding the species composition and environmental drivers of an ecosystem with little to no local anthropogenic stressors is critical in evaluating the broader impact of climate change on these ecosystems. Additionally, providing a more in-depth understanding of the ecosystems that provide the wealth of species harvested by Inuit in this region is critical to establishing the desired marine protections.

## Methods and materials

### Ethics statement

Data were collected by all authors in a collaborative effort. Research was conducted, which included photographs, visual estimates, and opportunistic collection of algae and invertebrates described in the methods below. The governments of Canada and Nunatsiavut granted all necessary permissions to conduct this research. No vertebrate sampling was conducted and therefore no approval was required by any Animal Care and Use Committee. Our data are publicly available at Data Dryad: https://doi.org/10.5061/dryad.0k6djhb61.

#### Expedition details

All data were collected as part of a larger research expedition to northern Labrador and Hudson Bay, Canada, aboard the Arctic icebreaker MV Polar Prince. An onboard laboratory with dissecting and compound microscopes aided in specimen identifications. Surveys were conducted in early summer (July12^th^ to 19^th^, 2022) and were intended to develop a baseline description of the species and habitats present in the shallowest (<15 m) nearshore areas of the fjord and its entrance, as well as the environmental factors likely accounting for species distributions.

### Study site

**Fig 1:**
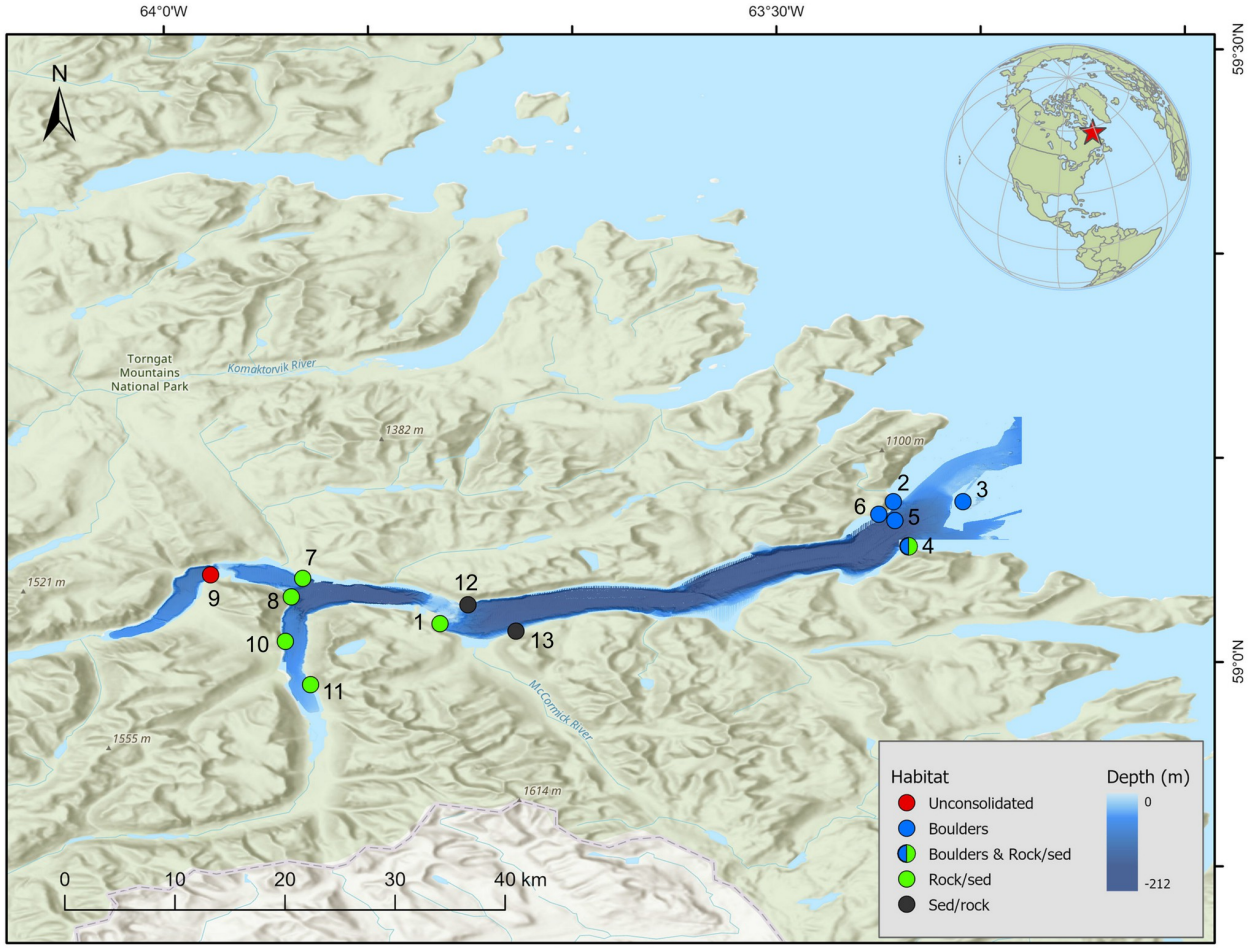
Sampling locations in Nachvak Fjord, Nunatsiavut, Northern Labrador. Basemap derived from GEBCO Compilation Group (2020) GEBCO 2020 Grid (doi:10.5285/a29c5465-b138-234de053-6c86abc040b9). Processing and assembly of the Global Self-consistent, Hierarchical, High-resolution Geography Database for shoreline data from [32].

Nachvak Fjord is a 45 km long by 2–4 km wide glacial trough that cuts through the heart of the Torngat Mountains, producing 1,000 m high sidewalls and 200 m deep basins that open to the Labrador Sea (Fig 1, [20, 30]). There are four successive basins in the fjord, becoming increasingly deeper from west to east, with mid-fjord water depth ranging from 90 to 210 m, separated by sills of depths of 10 to 180 m [21]. The average duration of the sea-ice cover is 6.6 mo yr^−1^ lasting from about mid-December to mid-July [22]. The landscape surrounding the fjord is covered by Arctic tundra and, although there are no glaciers, permanent ice fields remain during summer in the highest altitudes (around 1,500 m a.s.l.), with several rivers and streams flowing into the fjord, providing freshwater and sediments in summer and early autumn. There are no present-day human settlements in Nachvak Fjord or nearby, although historically this area was populated by ancient Inuit people and is used today for fishing and hunting grounds [31].

Nachvak Fjord is surrounded by the terrestrial Torngat Mountains National Park, which covers 9,700 km^2^ between Northern Québec and the Labrador Sea. It is a rugged region that is known for polar bears (*Ursus maritimus*), ringed seals (*Pusa hispida*), black bears (*Ursus americanus*), caribou (*Rangifer tarandus*) and a variety of polar birds and waterfowl such as black guillemot (*Cepphus* grille), glaucous gull (*Larus hyperboreus*), and common eider (*Somateria mollissima*). The park is accessible only by boat, charter plane, or helicopter during the summer and most visitors stay within the bear-fence-enclosed Torngat Mountains Base Camp and Research Station located outside the park on Saglek Fjord. Nachvak Fjord lies further north, away from the Nunatsiavut communities and can only be reached by boat, or by helicopter from Saglek Fjord.

### In-situ surveys: Macroalgae, megabenthic invertebrates, and fishes

Thirteen sites were surveyed ranging from the open waters just outside the fjord to the western and southern innermost arms of the fjord (Table 1). Characterization of subtidal communities was performed on SCUBA by deploying two transects of 25 m length per survey site. Transects were conducted parallel to shore, between 6 and 12 m depth.

**Table 1:** Metadata of survey sites sampled in Nachvak Fjord. Habitats: BOU = boulders, RwS = rocks with sediment, SwR = sediment with rocks, and UNC = unconsolidated, which included sand, gravel, or cobble. Temp. = temperature (C°), Sal. = salinity (ppt). See Fig 1 for Station ID locations.

Date	Station ID	Lattitude	Longitude	Habitat	Temp. (C°)	Sal. (ppt)	Depth (m)
12-Jul	1	59.0313	-63.7746	RwS	1.4	32.5	8
13-Jul	2	59.1310	-63.4044	BOU	1.9	32.4	8
13-Jul	3	59.1310	-63.3477	BOU	1.9	32.4	9
14-Jul	4	59.0944	-63.3922	BOU	1.9	32.4	8
14-Jul	5	59.1154	-63.4033	BOU	2.8	31.9	9
15-Jul	6	59.1205	-63.4165	BOU	1.0	32.3	12
16-Jul	7	59.0682	-63.8867	RwS	2.1	31.6	7
17-Jul	8	59.0531	-63.8959	RwS	0.7	32.4	11
17-Jul	9	59.0712	-63.9619	UNC	0.7	32.3	12
18-Jul	10	59.0167	-63.9006	RwS	0.8	32.4	10
18-Jul	11	58.9815	-63.8801	RwS	2.0	31.8	10
19-Jul	12	59.0466	-63.7519	SwR	1.4	32.5	10
19-Jul	13	59.0253	-63.7125	SwR	1.6	32.2	10

A line-point intercept methodology was used to quantify major components/categories of the habitats and their biogenic cover (e.g., erect macroalgae [including kelp], crustose coralline algae, bare rock, or unconsolidated sediment). The presence of each component was recorded every 20 cm on the transect line along two 25 m transects at each survey site. These data were then converted to percent cover for each transect. Macroalgae were identified to the lowest possible taxonomical level (genus or species). Owing to difficulties in distinguishing some species, we combined *Clathromorphum circumscriptum* and *C*. *compactum* into *Clathromorphum* spp. Similarly, we combined *Lithothamnion glaciale* and *L*. *tophiforme* into *Lithothamnion* spp.

For sessile and mobile megabenthic invertebrates, the number of individuals was estimated within a 1 m belt of either side of the transect line (50 m^2^) at each survey site (N = 2 transects). For colonial organisms (e.g., cnidarians, bryozoans, and tunicates) colonies, rather than individuals, were counted. Only non-cryptic megabenthic invertebrates > 1 cm were enumerated. Specimen identification was aided by *in situ* photographs and hand collections for identification in the laboratory. Identification of algae and megabenthic invertebrates were obtained from published literature [33–36], technical reports [37, 38], and websites [17, 39–41]. Megabenthic invertebrates were classified into five feedings guilds: active suspension feeders, passive suspension feeders, carnivores, herbivores, and detritivores [42].

For fish surveys, a scuba diver counted and sized all fishes within 1 m of either side of the 25 m transect lines (50 m^2^) at each survey site (N = 2 transects). Total fish lengths were estimated to the nearest cm. In addition, photographs were taken *in situ* to assist with species identification, and to document underwater coloration and habitat associations. Fish species identifications were obtained from published literature [43, 44]. The biomass of individual fishes was estimated using the allometric length-weight conversion: W = aTL^b^, where parameters a and b are species-specific constants, TL is total length in cm, and W is weight in grams. Length-weight fitting parameters were obtained from FishBase [45]. The sum of all individual weights and numerical densities was used to estimate biomass density by species.

Measurements of temperature, salinity, pH, dissolved oxygen, and chlorophyll-*a* were taken through the full depth of the water column at each survey location with a handheld YSI Pro DSS.

### Statistical analyses

For both megabenthic invertebrates and fishes, species diversity was calculated using the Shannon-Wiener diversity index [46]: H´ = Σ (*p*_*i*_ × ln *p*_*i*_), where *p*_*i*_ is the proportion of all individuals counted that were of species *i*. Pielou’s evenness was calculated as: J = H´/ln(S), where S is the total number of species present. Similarity percentages analysis (SIMPER) was used to determine the benthic categories most responsible for the percentage similarities in benthic cover within habitats using Bray-Curtis similarity analysis of hierarchical agglomerative group average clustering. A Bray–Curtis similarity matrix was created from percent cover of benthic categories and a Principal Coordinate Analysis (PCO) was used to visualize the distribution of transects in ordination space based on benthic cover. Vectors of the relative contribution and direction of influence of categories to the observed variation among sites (Spearman’s rank-order correlations ≥ 0.5) were projected onto the PCO plot.

Comparisons of megabenthic invertebrate and fish assemblage structure among habitats were investigated using permutation-based multivariate analysis of variance (PERMANOVA). A Bray–Curtis similarity matrix was created from abundance of benthic taxa, benthic functional groups, and fish species. Habitat was treated as a fixed factor. Interpretation of PERMANOVA results was aided using individual analysis of similarities (ANOSIM). All SIMPER, PCO, PERMANOVA, and ANOSIM analyses were performed using Primer 6.1.18.

To describe the patterns of benthic invertebrate and fish assemblage structure, and benthic invertebrate feeding guilds among locations and their relationship to environmental variables, we performed direct gradient analysis (redundancy analysis: RDA) using the ordination software CANOCO version 5.0 [47]. The RDA introduces a series of explanatory (environmental) variables and resembles the model of multivariate multiple regression, allowing us to determine what linear combinations of these explanatory variables determine the gradients. Predictor variables were centered and standardized to account for the wide differences in sampling units. Megabenthic invertebrate taxa and feeding guild abundances and fish taxa abundance were centered and log(x+1)-transformed for analyses. Predictor variables consisted of temperature (°C), salinity (ppt), depth (m), distance to the mouth of the fjord (km), habitat type, and the scores from the first two axes of the PCO of benthic cover.

## Results

### Benthic habitats

**Fig 2:**
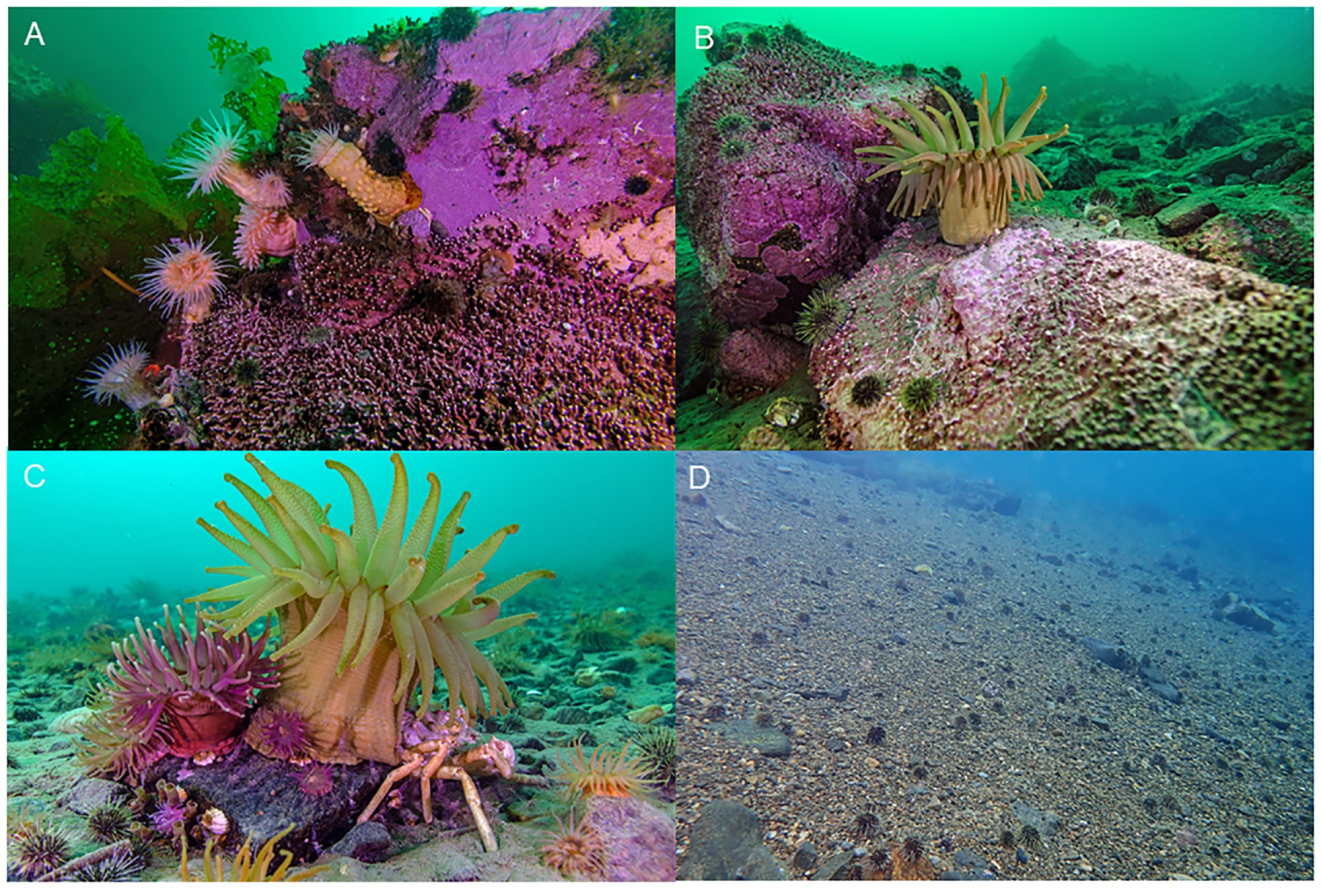
Benthic habitats observed in Nachvak Fjord. A. Boulder, B. Rocks with sediment, C. Sediment with scattered rocks, D. Unconsolidated sediment.

We identified four major benthic habitat types in Nachvak Fjord based on qualitative observations of the underlying substrate. These consisted of: (1) boulders = BOU, (2) rocks with sediment = RwS, (3) sediment with rocks = SwR, and (4) unconsolidated sediments = UNC, which included sand, gravel, or cobble (Fig 2). BOU habitat was dominated by the kelp species *Alaria esculenta* (35% of total cover), which contributed 49% to the similarity within this habitat. The coralline algae *Clathromorphum* spp. comprised an additional 17% of total benthic cover but only contributed 7% to group similarity. The kelp species *Saccharina latissima*, *Laminaria solidungula*, and *Hedophyllum nigripes* accounted for an additional 13%, 11%, and 7% of total benthic cover, respectively, and collectively contributed 36% to group similarity (Table 2). RwS habitat was dominated by bare rock (32%), *Lithothamnion* spp. (25%), and sediment (21%). Sediment accounted for 92% of the benthic cover in the UNC habitat. Similarly, the SwR habitat was dominated by sediment (81%) and bare rock (13%).

**Table 2:** Dominant algal taxa and benthic cover by major habitat type in Nachvak Fjord. Avg. (± sd) is average percent cover and one standard deviation. Percent contributions to average similarities within groups based on Bray-Curtis similarity matrices. % contrib. = percent contribution, % cum. contrib. = percent cumulative contribution.

**Boulders (BOU)**	**Avg. (sd)**	**% contrib.**	**% cum. contrib.**
**(Avg. similarity = 33.73)**			
*Alaria esculenta*	34.58 (16.42)	48.68	48.68
*Saccharina latissima*	12.71 (6.36)	18.84	67.52
*Laminaria solidungula*	10.75 (3.29)	9.75	77.27
*Hedophyllum nigriceps*	6.84 (2.56)	7.58	84.85
*Clathromorphum* spp.*	16.89 (2.42)	7.18	92.03
**Rocks w/sediment (RwS)**	**Avg. (sd)**	**% contrib.**	**% cum. contrib.**
**(Avg. similarity = 50.67)**			
Bare rock	32.16 (17.32)	34.18	34.18
*Lithothamnion* spp.*	25.04 (13.00)	25.65	59.82
*Clathromorphum* spp.*	14.80 (9.99)	19.72	79.54
Sediment	20.48 (8.28)	16.35	95.89
**Unconsolidated (UNC)**	**Avg. (sd)**	**% contrib.**	**% cum. contrib.**
**(Avg. similarity = 87.20)**			
Sediment	84.40 (80.00)	91.74	91.74
**Sediment w/rocks (SwR)**	**Avg. (sd)**	**% contrib.**	**% cum. contrib.**
**(Avg. similarity = 88.00)**			
Sediment	81.40 (76.13)	86.52	86.52
Bare rock	13.00 (9.33)	10.61	97.12

* *Clathromorphum* spp. includes *C*. *circumscriptum* and *C*. *compactum* and *Lithothamnion* spp. includes *L*. *glaciale* and *L*. *tophiforme*.

**Fig 3:**
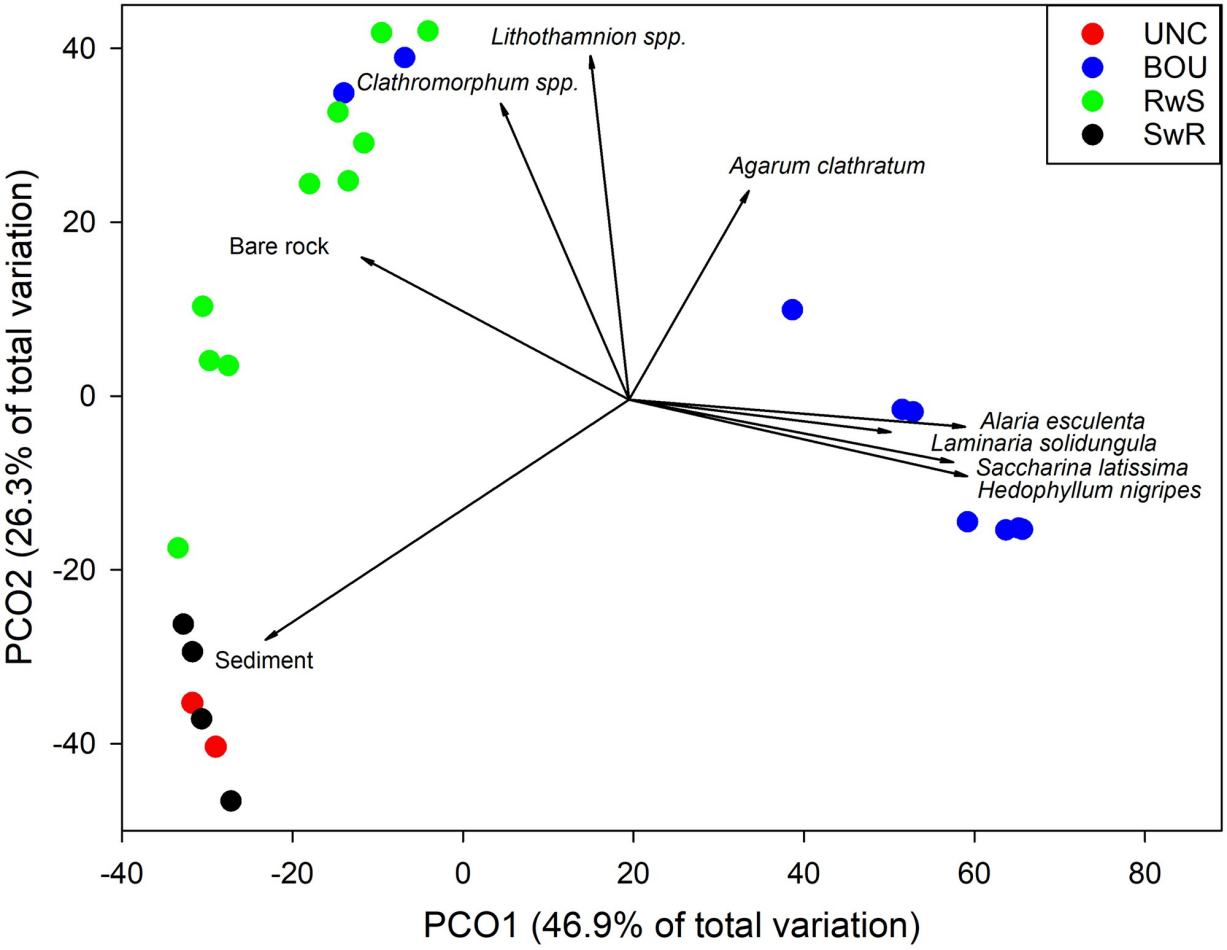
Principal coordinates analysis of benthic cover. Vectors are the relative contribution and direction of influence of taxa to the observed variation among sites (Spearman’s rank-order correlations ≥ 0.5). BOU—boulders, UNC—unconsolidated sediment, RwS—rocks with sediment, and SwR—sediment with rocks. *Clathromorphum* spp. includes *C*. *circumscriptum* and *C*. *compactum* and *Lithothamnion* spp. includes *L*. *glaciale* and *L*. *tophiforme*.

Based on benthic cover, BOU habitat was mostly separated in ordination space from the other habitat types due to laminarian kelp coverage, primarily *Alaria esculenta*, *Saccharina latissima*, *Hedophyllum nigripes*, and *Laminaria solidungula* (Fig 3). Encrusting coralline algae, mainly *Lithothamnion* spp. and *Clathromorphum* spp., were correlated with the RwS habitat, while sediment was most closely associated with the UNC and SwR habitats. PCO1 explained 46.9% of total variation in benthic cover with the major correlations being sediment and bare rock in the negative direction along PCO1, while the laminarian kelps *Alaria esculenta*, *Saccharina latissima*, *Laminaria solidungula*, and *Hedophyllum nigripes* comprised the major correlations with PCO1 in the positive direction (Table 3). PCO2 explained an additional 26.3% of total variation in benthic cover with sediment most highly correlated along the negative portion of PCO2 and *Clathromorphum* spp. and *Lithothamnion* spp. most highly correlated with the positive end of PCO2.

**Table 3:** Benthic cover correlations (Spearman’s rank-order correlations) from principal coordinates analysis.

Benthic cover	PCO1 (46.9%)	PCO2 (26.3%)
*Alaria esculenta* (winger kelp)	0.796	-0.067
*Hedophyllum nigriceps* (oarweed)	0.788	-0.173
*Saccharina latissima* (sugar kelp)	0.770	-0.157
*Laminaria solidungula* (Arctic kelp)	0.603	-0.072
*Desmarestia aculeata* (sea sorrel)	0.502	-0.078
*Palmaria palmata* (dulse)	0.451	-0.183
*Halosiphon tomentosus* (brown alga)	0.340	-0.113
*Phycodrys rubens* (sea oak)	0.307	-0.021
*Ptilota serrata* (northern sea fern)	0.307	-0.021
*Agarum cribosum* (sea colander kelp)	0.284	0.476
*Hildenbrandia* sp. (thalloid red algae)	0.181	0.293
*Semibalanus balanoides* (rock barnacle)	0.028	0.170
*Lithothamnion glaciale+ tophiforme* (encrusting coralline algae)	-0.082	0.797
*Cribrinopsis similis* (purple anemone)	-0.235	-0.139
*Clathromorphum circumscriptum+ compactum* (encrusting coralline algae)	-0.286	0.670
Bare rock	-0.639	0.325
Sediment	-0.862	-0.555

### Megabenthic invertebrates

**Fig 4:**
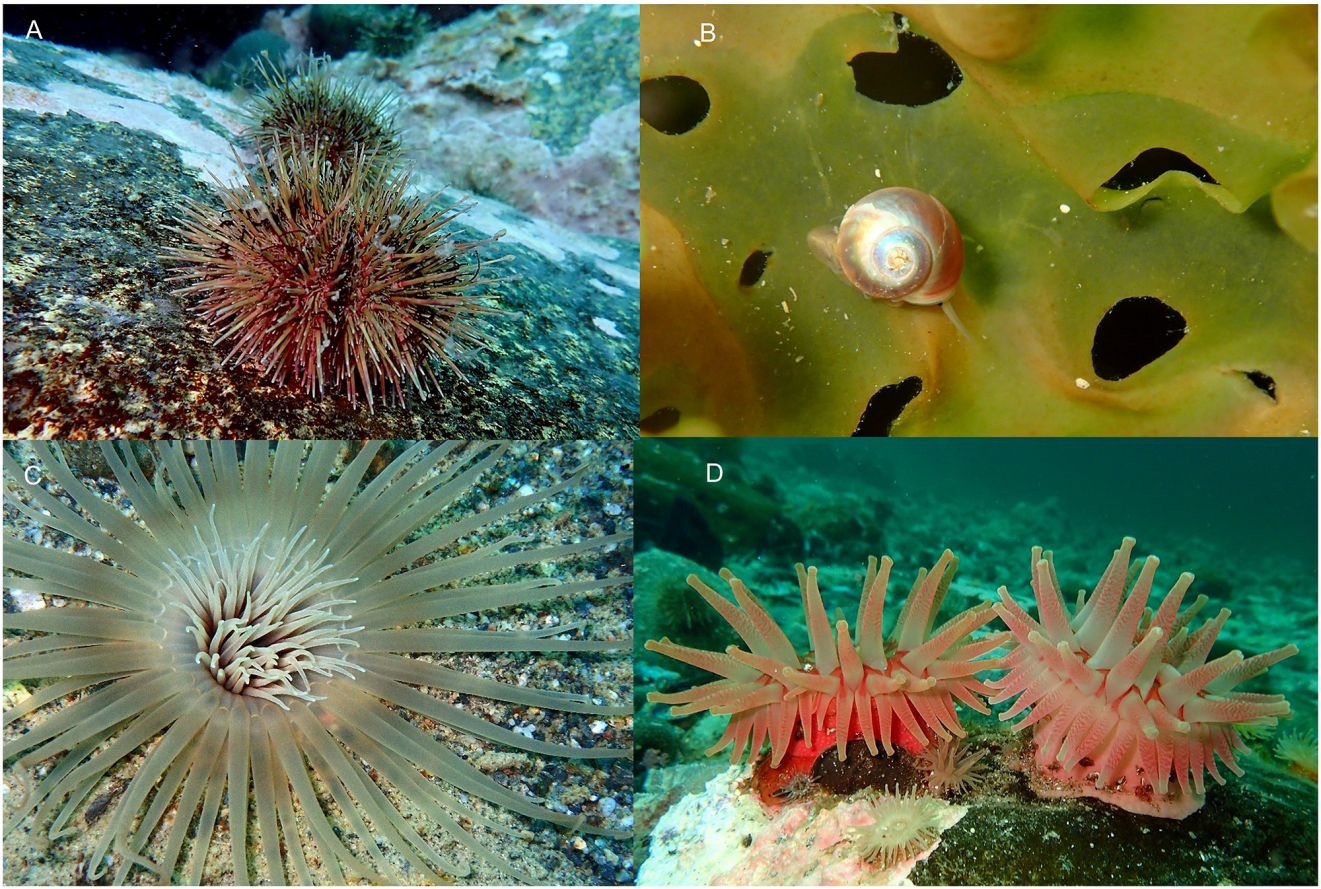
Common megabenthic invertebrates observed in Nachvak Fjord. A. *Strongylocentrotus droebachiensis* (green sea urchin), B. *Margarites helicinus* (spiral sea snail) on *Agarum clathratum* (sea colander kelp), C. *Pachycerianthus borealis* (tube anemone), D. *Cribrinopsis similis* (pink anemone).

A total of 44 megabenthic invertebrate taxa from 8 phyla, 12 classes, 22 orders, and 30 families were observed on quantitative transects in Nachvak Fjord during our surveys (S1 Table). The most species rich phylum was Echinodermata with 15 taxa, followed by Mollusca with 10, Cnidaria with 6, and Arthropoda with 5. The top ten taxa accounted for > 97% of total numerical abundance and were dominated by the green sea urchin *Strongylocentrotus droebachiensis*, which comprised 46% of the total numerical abundance, followed by the gastropod mollusk *Margarites helicinus*, accounting for an additional 41% of total abundance (Table 4, Fig 4, S2 Table). *Margarites helicinus* was largely confined to the kelp understory in the BOU habitat, while *Strongylocentrotus droebachiensis* was dominant in all habitats except BOU, where it was uncommon.

**Table 4:** Numerical abundance (number m^-2^) of the top ten megabenthic invertebrates among habitats. Values are means with one standard deviation in parentheses. UNC—unconsolidated sediment, BOU—boulders, RwS—rocks with sediment, SwR—sediment with rocks.

Taxa	UNC	BOU	RwS	SwR	Overall
*Strongylocentrotus droebachiensis* (green sea urchin)	24.00 (1.13)	3.24 (5.65)	30.16 (9.20)	28.30 (8.70)	19.68 (14.59)
*Margarites helicinus* (spiral sea snail)	-	49.11 (48.86)	0.03 (0.06)	-	17.69 (37.07)
*Pachycerianthus borealis*(tube anemone)	0.65 (0.49)	0.05 (0.13)	1.66 (1.78)	1.33 (1.42)	0.95 (1.42)
*Cribrinopsis similis* (pink anemone)	-	0.03 (0.09)	1.33 (1.18)	1.35 (1.40)	0.76 (1.10)
*Balanus balanus* (acorn barnacle)	—	0.94 (1.20)	0.52 (0.56)	0.70 (0.83)	0.66 (0.87)
*Mya truncata* (truncate softshell clam)	-	-	0.90 (1.78)	1.70 (1.90)	0.63 (1.43)
*Semibalanus balanoides* (rock barnacle)	-	0.08 (0.13)	0.82 (1.70)	1.46 (0.83)	0.59 (1.21)
*Sabella* sp. (feather duster worm)	1.25 (1.09)	<0.01 (0.01)	0.06 (0.09)	1.96 (1.83)	0.438 (1.021)
*Testudinalia testudinalis* (tortoise limpet)	0.23 (0.18)	0.05 (0.03)	0.41 (0.59)	0.26 (0.26)	0.24 (0.41)
*Didemnum albidum* (white crust tunicate)	-	-	0.52 (0.98)	0.03 (0.05)	0.21 (0.66)

RwS habitat had the highest richness of species per transect (17.0 ± 4.8), followed by SwR (16.7 ± 1.0), while BOU habitat had the lowest richness (6.8 ± 3.4) (Table 5). The number of individuals m^-2^ ranged from a high of 54.0 (± 44.9) in BOU habitat to 28.5 (± 0.7) in the UNC habitat. Both diversity and evenness were lowest in BOU habitat.

**Table 5:** Invertebrate assemblage characteristics among habitat types in Nachvak Fjord. Values are means and standard deviation in parentheses.

Habitat	Species	num. m^-2^	Diversity	Evenness
Unconsolidated (UNC)	8.50 (0.71)	28.50 (0.71)	0.62 (0.03)	0.29 (0.01)
Boulders (BOU)	6.78 (3.38)	54.00 (44.95)	0.35 (0.45)	0.17 (0.20)
Rocks w/sediment (RwS)	17.00 (4.78)	38.20 (10.02)	0.92 (0.34)	0.33 (0.11)
Sediment w/rocks (SwR)	16.75 (0.96)	38.25 (13.33)	1.01 (0.30)	0.36 (0.11)

**Fig 5:**
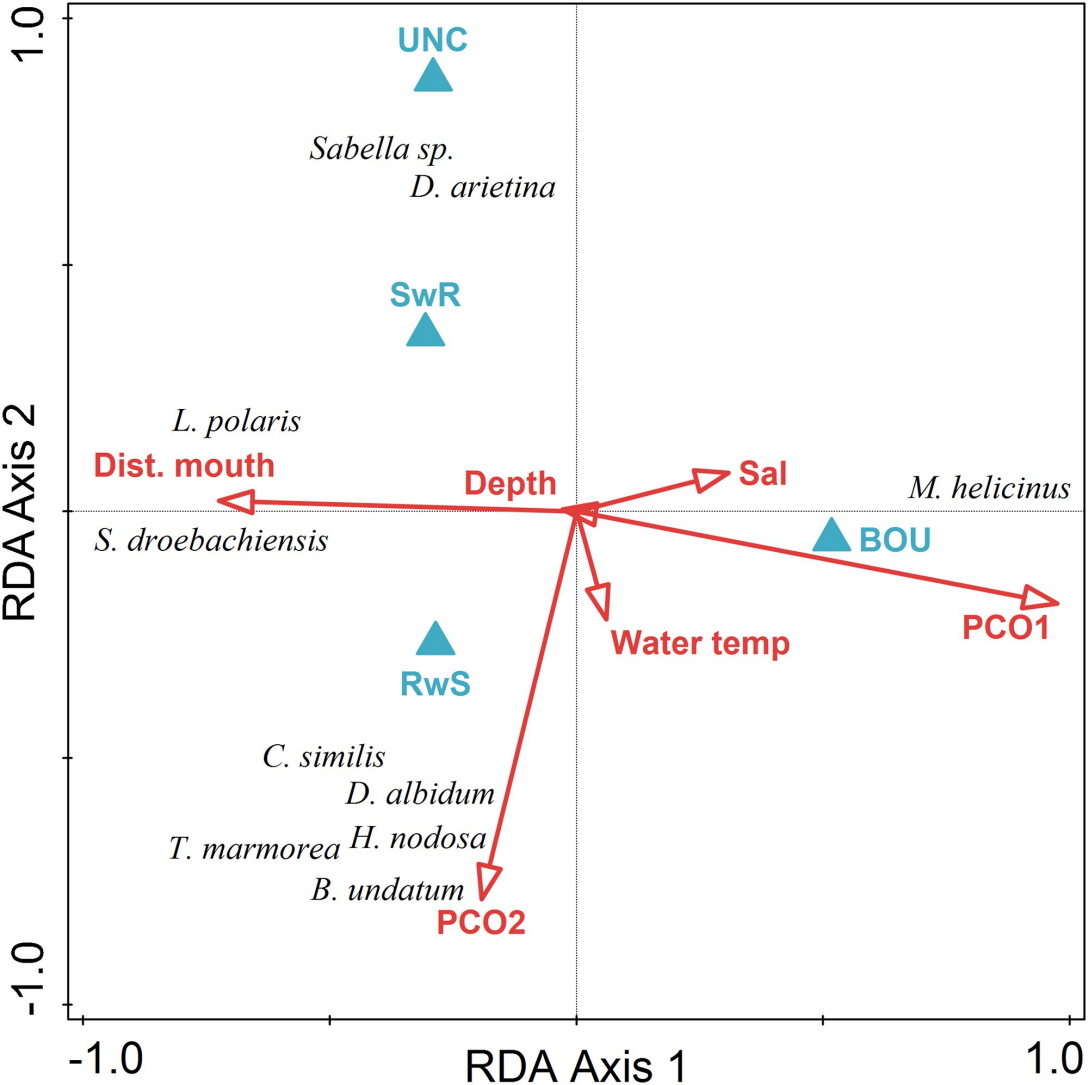
Triplot of results of redundancy analysis on benthic taxa abundance by transect with biotic and abiotic variables (PC1, PC2, depth, salinity, water temperature, habitat type, distance to mouth of fjord). Predictor variables were centered and standardized and megabenthic invertebrate taxa abundance was centered and log(x+1)-transformed for analysis.

There was a significant difference in megabenthic invertebrate assemblage structure among habitats based on numerical abundance (PERMANOVA F_3,24_ = 6.605, p = 0.001). The BOU habitat was significantly different from all other habitats, and these other habitats were not significantly different from one another (Table 6). Based on numerical abundance of megabenthic invertebrate taxa, habitats were well separated in ordination space with BOU and RwS habitats distinct from each other and SwR and UNC habitats in closer proximity to one another (Table 7, Fig 5). The first two axes of the RDA explained 76% of community-level variance and 88% of the community and environmental variables relationship. PCO1, a proxy for benthic cover, explained 66.4% to the variability in community structure and was correlated with boulder habitat. PCO2 was orthogonal to PCO1, contributed an additional 7.5% of the variability and was correlated with RwS habitat.

**Table 6:** Comparisons of megabenthic invertebrate assemblage structure among habitats based on numerical abundance (number m^-2^). Results of permutation-based multivariate analysis of variance (PERMANOVA) and pair-wise analysis of similarities (ANOSIM).

Source	df	MS	Pseudo-F	P(perm)
Habitat	3	9319.9	6.605	0.001
Residuals	21	1411.0		
Total	24			
Groups*	R statistic	P		
RwS, BOU	0.662	0.001		
BOU, UNC	0.556	0.018		
BOU, SwR	0.454	0.011		
RwS, UNC	0.087	0.288		
UNC, SwR	0.071	0.400		
RwS, SwR	-0.033	0.500		

*BOU—boulders, UNC—unconsolidated sediment, RwS—rocks with sediment, and SwR—sediment with rocks.

**Table 7:** (a) Results of redundancy analysis (RDA) on ln(x+1)‐transformed benthic taxa abundance (num. m^-2^) by transect. (b) Conditional effects of Monte‐Carlo permutation results on the RDA.

Statistic	Axis 1	Axis 2	Axis 3	Axis 4
Eigenvalues	0.697	0.064	0.044	0.027
Explained variation (cumulative)	69.700	76.050	80.420	83.160
Pseudo-canonical correlation	0.989	0.930	0.864	0.905
Explained fitted variation (cumulative)	80.920	88.290	93.370	96.540
Name	% explained	pseudo-F	P	P(adj)
PCO1	66.4	45.5	0.002	0.006
PCO2	7.5	6.3	0.002	0.006
UNC	3.2	3.0	0.004	0.009
Dist. to fjord mouth	3.3	3.4	0.002	0.006
SwR	2.5	2.8	0.014	0.025
Water temp. (°C)	1.8	2.2	0.020	0.030

The small, mainly kelp-associated snail, *Margarites helicinus* was strongly correlated with PCO1 and BOU habitat. The mottled chiton *Tonicella marmorea*, two anemones *Cribrinopsis similis* and *Hormathia nodosa*, a colonial tunicate *Didemnum albidum*, and the waved whelk *Buccinum undatum* were correlated with PCO2 and RwS habitat. Sabellid tube worms (*Sabella* sp.) were most closely associated with UNC and SwR habitats.

Two species of sea urchin were observed on transects in the Nachvak Fjord. S*trongylocentrotus droebachiensis* and *S*. *pallidus*. S*trongylocentrotus droebachiensis* was by far the most common, occurring on 76% of transects with an average density of 19.68 indiv. m^-2^ (± 14.59 sd), while *S*. *pallidus* was observed in much lower abundances (0.02 indiv. m^-2^ ± 0.04). Sea urchins were absent from the six transects dominated by kelp. Anemones were extremely common outside kelp dominated habitats and were a major component of the megabenthic invertebrate community. Observed anemone species included: *Cribrinopsis similis*, *Aulactinia stella*, *Pachycerianthus borealis*, *Hormathia nodosa*, *Urticina felina* and *Stomphia coccinea*. Other relevant megabenthic invertebrates found in our surveys include the acorn barnacle *Balanus balanus* and rock barnacle *Semibalanus balanoides*, the tortoise limpet *Testudinalia testudinalis*, the seastars *Leptasterias polaris*, *L*. cf. *littoralis*, *Crossaster papposus* and *Solaster endeca*, and the sea cucumbers *Psolus fabricii* and *Cucumaria frondosa*. The polar shrimp *Lebbeus polaris* was observed in almost every transect.

**Fig 6:**
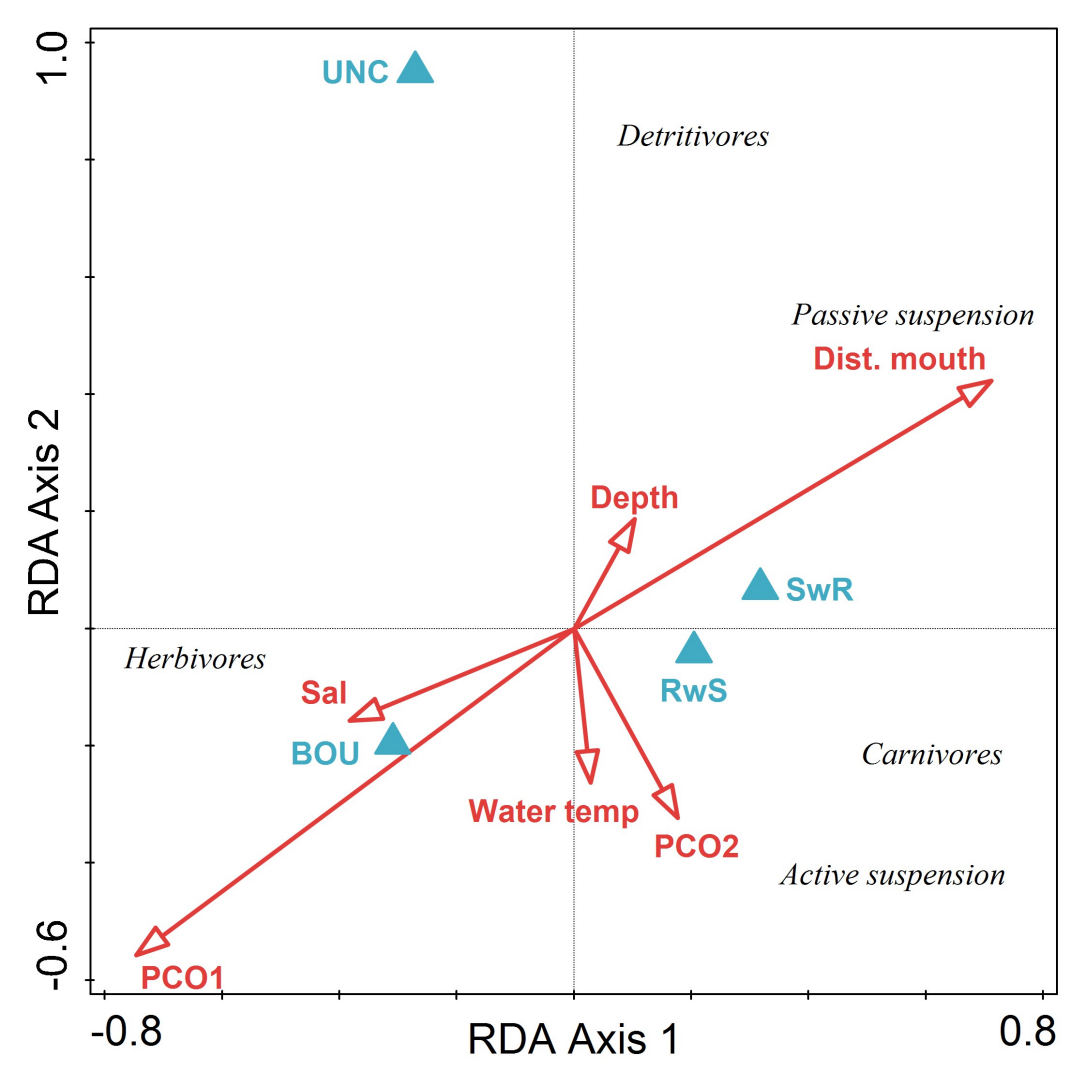
Triplot of results of redundancy analysis on numerical abundance (num. m^-2^) of megabenthic invertebrate feeding guilds by transect with biotic and abiotic variables (PC1, PC2, depth, salinity, temperature, habitat type, distance to mouth of fjord). Predictor variables were centered and standardized, and megabenthic invertebrate feeding guild abundance data were centered and log(x+1)-transformed for analysis.

There was no significant difference in megabenthic invertebrate assemblage structure among habitats based on feeding guilds (PERMANOVA F_3,24_ = 1.740, p = 0.106). SwR and RwS habitats clustered together in ordination space, while BOU and UNC habitats were well separated along RDA Axis 2 (Table 8, Fig 6). The first two axes of the RDA explained 54% of community-level variance and 85% of the community and environmental variables relationship. PCO1, a proxy for benthic cover, explained 25% of the variability in community structure and was correlated with BOU habitat. UNC habitat accounted for an additional 19% of the community structure, followed by distance to the mouth of the fjord at 6%. Carnivores and active suspension feeders were correlated with SwR and RwS habitats. Detritivores were most closely correlated with UNC habitat and herbivores were most closely correlated with BOU habitat.

**Table 8:** (a) Results of redundancy analysis (RDA) on ln(x+1)‐transformed megabenthic invertebrate feeding guild abundance (num. m^-2^) by transect. (b) Conditional effects of Monte‐Carlo permutation results on the RDA. Predictor variables were centered and standardized and megabenthic invertebrate feeding guild abundance was log(x+1)-transformed prior to analysis.

Statistic	Axis 1	Axis 2	Axis 3	Axis 4
Eigenvalues	0.33	0.21	0.06	0.04
Explained variation (cumulative)	33.14	54.31	60.15	63.89
Pseudo-canonical correlation	0.79	0.91	0.83	0.55
Explained fitted variation (cumulative)	51.83	84.94	94.08	99.92
Name	% explained	pseudo-F	P	P(adj)
PCO1	25.1	7.7	0.006	0.027
UNC	19.4	7.7	0.002	0.018
Dist. to fjord mouth	5.7	2.4	0.064	0.192

UNC—unconsolidated habitat.

### Fishes

A total of 13 fish taxa from 7 families were observed in Nachvak Fjord during our surveys (Table 9). Cottidae was the most speciose family with five different species observed on transects. The ribbed sculpin, *Triglops pingelli*, was the most numerically abundant fish species, accounting for 56% of total numerical abundance and occurring on 36% of transects. The shorthorn sculpin, *Myoxocephalus scorpius*, was the second most numerically abundant species, accounting for 18% of total numerical abundance and was observed on 52% of transects. *Myoxocephalus scorpius* accounted for 61% of total biomass, while the Greenland cod, *Gadus ogac*, comprised an additional 30%.

**Table 9:** Fish species observed in Nachvak Fjord. num. m^-2^ = number of individuals per m^-2^, g m^-2^ = grams per m^-2^, Freq % = percent frequency of occurrence. Reported sizes (cm) are means, with minimum and maximum sizes in parentheses.

Family	Taxa	num. m^-2^	g m^-2^	Freq %	N	Size (cm)
Agonidae	*Aspidophoroides olrikii*	-	-	-	1	15.0
Cottidae	*Myoxocephalus quadricornis*	<0.001 (0.004)	0.065 (0.325)	4.0	1	22.0
Cottidae	*Myoxocephalus scorpius*	0.038 (0.077)	23.198 (75.147)	52.0	47	31.5 (18–45)
Cottidae	*Myoxocephalus scorpioides*	-	-	-	1	45.0
Cottidae	*Triglops nybelini*	0.008 (0.018)	0.015 (0.035)	20.0	55	4.3 (3–8)
Cottidae	*Triglops pingelli*	0.117 (0.272)	0.076 (0.179)	36.0	95	4.1 (3–10
Cyclopteridae	*Eumicrotremus spinosus*	0.008 (0.017)	0.005 (0.012)	20.0	10	2.4 (2–3)
Gadidae	*Boreogadus saida*	0.004 (0.010)	1.804 (4.416)	16.0	5	37.0 (31–45)
Gadidae	*Gadus ogac*	0.026 (0.043)	10.941 (27.505)	36.0	31	28.7 (10–65)
Liparidae	Liparidae unidentified	<0.001 (0.004)	0.064 (0.320)	4.0	1	15.0
Pholidae	*Pholis fasciata*	0.004 (0.008)	0.166 (0.366)	20.0	5	22.0 (17–25)
Stichaeidae	*Lumpenus fabricii*	<0.001 (0.004)	0.010 (0.050)	4.0	1	16.0
Stichaeidae	*Stichaeus punctatus*	<0.001 (0.004)	0.007 (0.034)	4.0	1	12.0

RwS habitat had the highest richness of species per transect (2.9 ± 1.5), while BOU habitat had the lowest richness (1.3 ± 1.1) (Table 10). However, biomass was 2.9 times higher in BOU habitat compared to RwS, 22.5 times higher compared to UNC habitat, and 53.4 times higher compared to SwR habitat. The number of individuals m^-2^ ranged from a high of 0.99 (± 0.01) in UNC habitat to 0.09 (± 0.14) in BOU habitat. Both diversity and evenness were lowest in UNC habitat, which was dominated by the small, ribbed sculpin *Triglops pingelli*.

**Table 10:** Fish assemblage characteristics among habitat types in Nachvak Fjord. Values are means with one standard deviation in parentheses.

Habitat	Species	num. m^-2^	g m^-2^	Diversity	Evenness
UNC	2.00 (0.01)	0.99 (0.01)	3.22 (3.48)	0.10 (0.01)	0.14 (0.01)
BOU	1.33 (1.12)	0.09 (0.14)	72.38 (138.80)	0.29 (0.45)	0.30 (0.45)
RwS	2.90 (1.52)	0.19 (0.17)	24.55 (42.12)	0.73 (0.50)	0.62 (0.36)
SwR	2.25 (0.96)	0.12 (0.07)	1.36 (0.94)	0.59 (0.45)	0.61 (0.41)

BOU—boulder habitat, UNC—unconsolidated habitat, RwS—rocks with sediment, SwR—sediment with rocks

**Fig 7:**
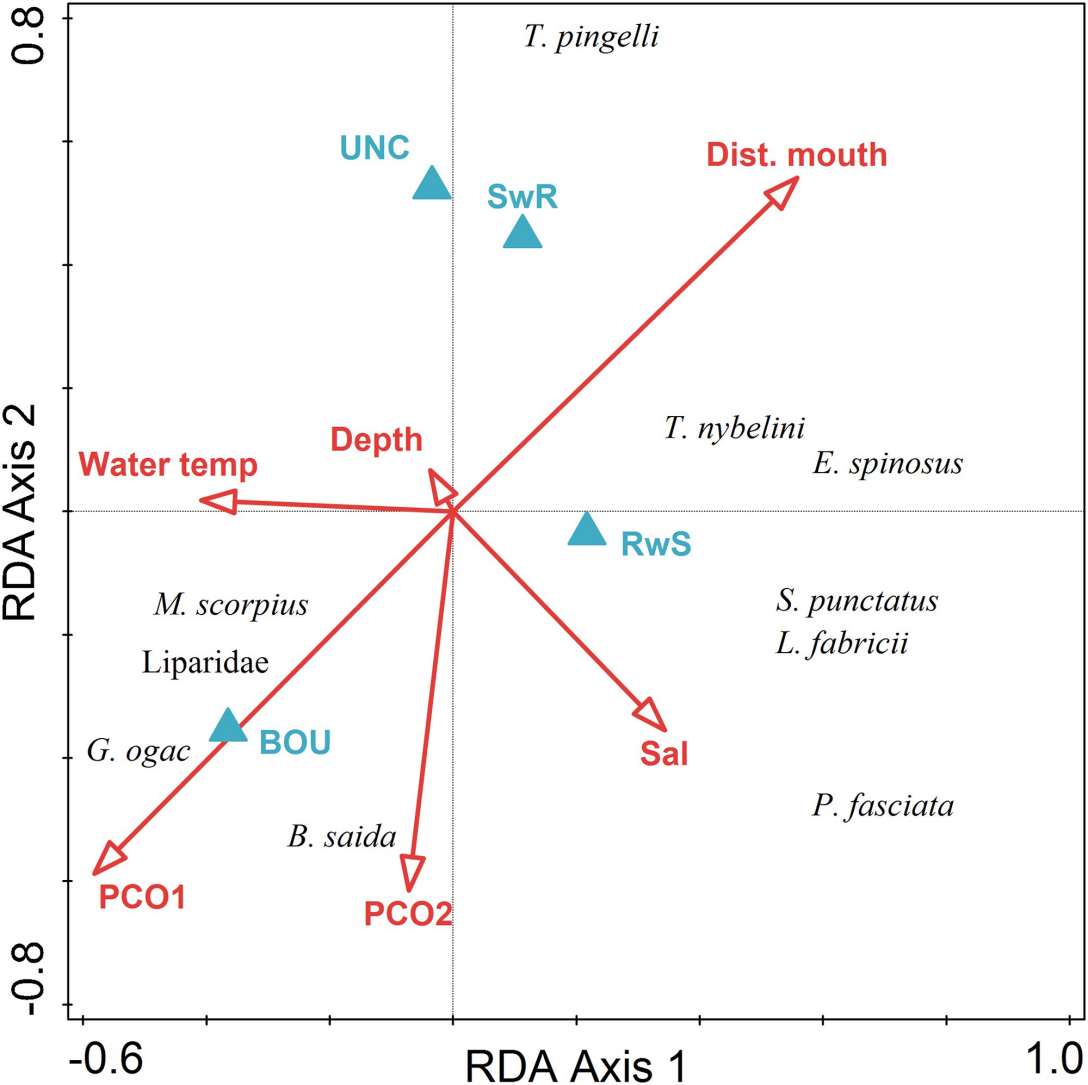
Triplot of results of redundancy analysis on fish taxa abundance (num. m^-2^) by transect with biotic and abiotic variables (PC1, PC2, depth, salinity, temperature, habitat type, distance to mouth of fjord). Predictor variables were centered and standardized, and fish taxa abundance data were centered and log(x+1)-transformed for analysis.

There was a significant difference in fish assemblage structure among habitats based on numerical abundance (PERMANOVA F_3,24_ = 6.605, p = 0.001). The BOU habitat was significantly different from SwR and UNC habitats, and these habitats were not significantly different from one another (Table 11). Based on numerical abundance of fishes, the first two axes of the RDA explained 28% of assemblage-level variance and 53% of the fish assemblage and environmental variables relationship (Table 12, Fig 7). BOU habitat explained 11% of the variability in fish assemblage structure, followed by distance to the mouth of the fjord (10.4%), and PCO1 (10.4%). The ribbed sculpin, *Triglops pingelli* was strongly correlated with UNC and SwR habitats and distance to the mouth of the fjord. *Boreogadus saida* and *Pholis fasciata* were correlated with BOU habitat.

**Table 11:** Comparisons of fish assemblage structure among habitats based on numerical abundance (num. m^-2^). Results of permutation-based multivariate analysis of variance (PERMANOVA) and pair-wise analysis of similarities (ANOSIM).

Source	df	MS	Pseudo-F	P(perm)
Habitat	3	7987.3	2.580	0.002
Residuals	19	3094.4		
Total	22			
Groups*	R statistic	P		
UNC, SwR	0.625	0.067		
BOU, UNC	0.614	0.028		
BOU, SwR	0.540	0.006		
RwS, UNC	0.248	0.091		
RwS, BOU	0.157	0.054		
RwS, SwR	0.023	0.373		

*BOU—boulders, UNC—unconsolidated sediment, RwS—rocks with sediment, and SwR—sediment with rocks.

**Table 12:** (a) Results of redundancy analysis (RDA) on ln(x+1)‐transformed fish taxa abundance (num. m^-2^) by transect. (b) Conditional effects of Monte‐Carlo permutation results on the RDA.

Statistic	Axis 1	Axis 2	Axis 3	Axis 4
Eigenvalues	0.16	0.11	0.10	0.07
Explained variation (cumulative)	16.12	27.60	37.19	44.11
Pseudo-canonical correlation	0.95	0.80	0.87	0.80
Explained fitted variation (cumulative)	31.04	53.13	71.59	84.90
Name	% explained	pseudo-F	P	P (adj)
BOU	11.1	2.6	0.008	0.024
Dist. mouth	10.4	2.4	0.006	0.024
PCO1	10.4	2.4	0.006	0.024

## Discussion

Detailed studies of nearshore benthic marine macrofauna and flora for the Canadian Arctic are relatively scarce [19, 22]. To our knowledge, no previous peer-reviewed studies on shallow water habitats have been conducted in Northern Labrador. This area has long been important to the Nunatsiavut people and our findings complement the existing Inuit Knowledge from the region by providing characterization of the underwater ecosystem. The remoteness and low human population density of Arctic regions are often associated with relatively intact marine communities [48, 49], however, these areas are now at the forefront of climate change, and ocean warming will likely lead to drastic changes in Arctic ecosystems [50, 51]. This study documents the shallow subtidal communities of Nachvak Fjord, serving as a first baseline for the region and providing a benchmark against which to evaluate change. However, there have likely been substantial changes to this ecosystem over the past several decades due to climate change, which must be considered in any evaluation.

We observed four major habitat types within the fjord, which harbored distinct megabenthic invertebrate and fish assemblages. Kelp cover, primarily *Alaria esculenta*, was high on boulders outside Nachvak Fjord, with few megabenthic invertebrates present, except for the gastropod mollusk *Margarites helicinus*. Large-bodied fishes, such as Greenland cod (*Gadus ogac*) and Arctic cod (*Boreogadus saida*), dominated fish biomass in this habitat on the outside of the fjord, but were not found in high abundances further inside the fjord. Inside the fjord was devoid of kelp and encrusting coralline algae dominated the rock with sediment habitat. Coralline algae-dominated rocky bottoms and unconsolidated habitats. These habitats also harbored high abundances of sea urchins. The two contrasting situations of kelp without sea urchins and coralline algae with sea urchins, indicate that sea urchin abundance may be the main driver limiting kelp development inside Nachvak Fjord. In fact, the only kelp species that was found thriving at high sea urchin densities in Nachvak is *Agarum clathratum*, a species with allelochemicals that deters grazing [52, 53]. This finding has also been reported for other parts of the eastern Canadian Arctic [14]. However, many factors can contribute and intertwine to control urchin-kelp dynamics, several of which may be concurrently at play in Nachvak Fjord.

The presence of kelp and that of sea urchins are often mutually exclusive, as sea urchin grazing pressure is the main biological interaction limiting the abundance of kelp in eastern Canada [54–57]. While the existence of sea urchin barrens has been previously noted in southern Labrador [58, 59], surveys conducted in the northern Labrador Torngat region outside of the fjords were dominated by kelp [14]. Kelp forest dominance in outer Nachvak Fjord agrees with suggestions that the Torngat area is a boundary between kelp-dominated versus sea urchin-dominated habitats in the Canadian Arctic [14].

Sea urchin abundance in Nova Scotia, south of Labrador, seems to be limited by disease outbreaks of the amoeba pathogen *Paramoeba valens*, whose development is only possible during warming events [60]. Nachvak Fjord may currently not be experiencing this kind of sea urchin regulation, as it is farther north and more directly affected by the cold northern current coming from the Arctic [4, 61–63], and does not experience the high summer seawater temperatures found further south in Nova Scotia. Also, Nachvak Fjord lacks the presence of the North American lobster (*Homarus americanus*), a sea urchin predator reported to regulate sea urchin populations in eastern Canada [64, 65]. Given this, we did not observe a biotic interaction capable of limiting the abundance of the dominant sea urchin *Strongylocentrotus droebachiensis* in Nachvak. However, it is possible that the range of *H*. *americanus* may change with warming water regimes over the coming decades and potentially cause shifts in marine communities. Similarly, *P*. *valens* may affect communities further north as current regimes shift and waters warm. Our data therefore serves as an important baseline against which to monitor such community shifts.

The presence of kelp can also be impacted by the interaction between biotic and abiotic factors and several of these may contribute to the pattern of habitat distribution we observed in Nachvak Fjord. The outer areas of the fjord are more exposed to wave action, which is known to reduce the rate of movement and grazing by sea urchins [66, 67]. Additionally, the inner portions of the fjord has less surge and more freshwater inputs, resulting in higher siltation rates. These processes can diminish kelp productivity and growth [68], leaving kelp more susceptible to the effects of grazers. In fact, Bonsell and Dunton [69] found a positive correlation between cover of encrusting coralline algae and freshwater inputs in a high Arctic region of the Beaufort Sea. However, when sampling in western Greenland, Schoenrock et al. [70] did not find a clear pattern in the distribution of kelp beds and coralline algal-dominated communities in less exposed zones. It is notable that their survey sites did not follow along a fjord gradient but were interspersed between several islands in rather sheltered environments.

At the regional scale of the Canadian Arctic and Greenland, sea ice condition has been found to be the major environmental driver accounting for the distribution and biomass of kelp [14, 71], with higher species diversity and seaweed biomass in places that had the most open water days with exposed sea surface (ice-free). Nevertheless, this geographical pattern did not apply to Northern Labrador coasts sampled in previous studies, which despite hosting the most open water days of all the Canadian Arctic were found to be dominated by sea urchins and with little to no kelp [14]. However, the sites examined in previously studies were less exposed, and it is possible that the more exposed sites we sampled at Nachvak, which had high kelp cover, are characterized by earlier ice break-up and therefore have more ice-free days than those inside the less exposed areas of the fjord.

Change in the dominant substrate type could also account for the habitat differences found along Nachvak Fjord. Boulders were the dominant substrate at outer fjord sites, and their extension decreased moving westward into the fjord. This substrate was replaced by rocks with sediment, sediment with rocks, and unconsolidated sediments further inside the fjord. However, some of the largest kelp forests in the eastern Canadian Arctic thrive over unconsolidated substrate with gravel or scattered cobbles [14]. Therefore, other factors must be contributing to the habitat differences observed.

Rocks with sediment habitats devoid of kelp in the middle and inner reaches of Nachvak Fjord showed more diverse megabenthic invertebrate assemblages than the outer fjord kelp beds, with a high richness of anemones, whelks, and sea stars even though, at times, the habitats were inundated by sea urchins. This situation contrasts with the widespread theory that kelp forests are biodiversity hot spots [72–74]. The outer portion of Nachvak Fjord held an amazingly high density of kelp, which could be preventing the settlement and growth of large suspension feeders and their predators by occupying available space. These beds showed a high diversity of kelp species and red erect macroalgae (*Ptilota serrata*, *Euthora cristata*, *Fimbrifolium dichotomun*, *Phycodrys fimbriata* and others) and encrusting corallines (*Lithothamnion*, *Clathromorphum*) in the basal layer, but these macroalgae were mainly concentrated in the patches with low kelp density. The gastropod mollusk (*Margarites helicinus*) was the dominant megabenthic invertebrate in the kelp forests; however, this habitat likely hosts a high diversity of cryptic species such as small fishes, amphipods, and other megabenthic invertebrates not seen in the visual censuses.

Unconsolidated sediment habitats inside the fjord hosted a low number of species, although sabellid tube worms and the burrowing bivalves, *Mya truncata* and *Hiatella arctica*, were extremely common. This is consistent with the benthic habitat findings in Okak Bay, a nearby fjord in Central Labrador [19], despite the differences in collection methodologies. Carpenter et al. [19] used box corers and ROV transects to distinguish kelp beds, bedrock, boulders, and three different sedimentary habitat types in relation to sediment granulometry. While habitat classifications were somewhat similar, taxa assemblages were quite different, which can partially be explained by differences in sampling methodologies. In the kelp beds, our visual surveys found complex multispecies kelp communities whereas Carpenter et al. [19] reported an environment dominated by *Agarum clathratum*. Likewise, we found the rocks with sediment and sediment with rocks habitat to have the most species rich and diverse megabenthic invertebrate assemblage whereas in Okak Bay, the unconsolidated habitat consisting of gravelly sand was the most diverse, dominated by numerous bivalve species, polychaetes from the family Lumbrineridae, amphipods, and the green sea urchin *Strongylocentrotus droebachiensis* [19]. Despite the use of different sampling methodologies, they both provide further insight into Labrador fjord ecosystems.

Nearshore marine fish diversity and abundance in Nachvak Fjord was low overall and is likely limited by harsh environmental conditions (e.g., low water temperature, ice cover). Habitat explained much of the variation in fish assemblage structure with boulder habitat harboring the greatest biomass owing to the associated high habitat complexity and kelp cover. Most fish species were small except for three taxa of sculpins (*Myoxocephalus* spp.), Arctic cod (*Boreogadus saida*), and Greenland cod (*Gadus ogac*). A previous list of fishes for Nachvak and Saglek fjords collected using a variety of methods included 13 marine species, plus 5 additional species probably occurring in Nachvak Fjord [75]. Of the species listed, we observed 9 on our visual surveys and added 4 additional species to the list (*Triglops pingelli*, *T*. *nybelini*, *Eumicrotremus spinosus*, and *Pholis fasciata*). Our lower number of observed species reflects the bias associated with visual surveys, as well as several species that may have been misidentified in previous surveys. Arctic char (*Salvelinus alpinus*) were the most numerous species captured in previous studies using nets and while they were not observed on visual transects, we noted them at stream mouths and on our baited camera systems.

Visual surveys of fishes in kelp forests and rhodolith beds in southwestern Greenland only recorded three species of fishes (*Gadus morhua*, *Leptocottus armatus*, and *Myoxocephalus scorpius*) [70], which partially reflects the sampling biases of visual surveys but also the rarity of fishes in this region. While the current nearshore fish assemblage of Nachvak Fjord is depauperate, the marine ecosystem has evolved under these conditions. As marine species redistribute in a warming climate, changes in the spatial overlap between species and their predators, prey and competitors are likely to alter ecological interactions and drive changes in abundance, with impacts cascading throughout the entire food webs [76, 77].

Overall, our study describes the composition and distribution of shallow benthic habitats in a Northern Labrador fjord and explores the factors accounting for their distribution. There is a clear habitat gradient from outer to inner sites along the fjord with strong evidence pointing to sea urchin density as a key biotic factor in habitat distribution. Since both kelps and sea urchins are reported to depend on abiotic environmental constraints expected to change with warming and given the rapid changes the Arctic has experienced during the past decades, this study provides an important baseline for potential continuous regional survey efforts. Benthic ecosystem structure is largely determined by the interplay between environmental variables (e.g., temperature, ice cover, pH, salinity, irradiance, siltation), which change seasonally as well as at ecological-time scales, and variables operating at longer time scales (e.g., depth, position in the fjord, substrate, distance from river inputs). In our opinion, how the first group of variables and the interactions established with biotic factors (e.g., sea urchin grazing, photosynthetic activity, kelp growth) will evolve in the next decades will define the persistence of, or shifts in, the composition and distribution of nearshore habitats in Nachvak Fjord and in the Arctic in general. The outcome of these processes may ultimately change the relationship with nature, lifestyles, and traditional activities of Nunatsiavut communities and all Inuit groups in the Arctic.

Labrador Inuit Knowledge regarding harvested marine organisms in the fjords and coastal regions of the Nunatsiavut Marine Region includes the abundance of sea urchins, sea stars, waterfowl, fishes, seals, and whales in these waters. The results of this study complement the Labrador Inuit knowledge by contributing new information on some species and identifying the habitats they utilize. The shallow subtidal fish, megabenthic invertebrate, and kelp communities observed during our study also support many culturally and biologically important marine-associated animals including seabirds, seals, otters, and polar bears. The near pristine state of these waters, along with their cultural and ecological importance to Labrador Inuit, supports the desire of the Inuit to protect this marine environment against current and future anthropogenic threats. The results of this study provide an in-depth analysis of the habitats and ecology of the underwater realm of the Nunatsiavut fjords, lending additional information towards understanding what there is to be protected.

## Supporting information

S1 Table Megainvertebrates observed on quantitative transects in Nachvak Fjord during our surveys in 2022. (PDF)

S2 Table Numerical abundance (number m-2) of megainvertebrates among habitats. Values are means with one standard deviation in parentheses. BOU = boulders, RwS = rocks with sediment, SwR = sediment with rocks, and UNC = unconsolidated, which included sand, gravel, or cobble.
(PDF)
