# Supplementary material for: Shallow subtidal marine benthic communities of Nachvak Fjord, Nunatsiavut, Labrador: A glimpse into species composition and drivers of their distribution
Source: PLoS One. 2023 Nov 9;18(11):e0293702. doi: 10.1371/journal.pone.0293702 (PMC10635441; doi:10.1371/journal.pone.0293702)
Supplement: S1 Table — (PDF) [file pone.0293702.s001.pdf]

S1 Table. Megainvertebrates observed on quantitative transects in Nachvak Fjord during our surveys in 2022.

Phylum	Class	Order	Family	Taxa	Feeding guild
Annelida		Sipuncula	Golfingidae	*Golfingia (Golfingia) margaritacea*	Detritivores
Annelida	Polychaeta	Sabellida	Sabellidae	*Sabella* sp.	Passive suspension feeder
Annelida	Polychaeta	Terebellida	Pectinariidae	*Pectinaria gouldii*	Detritivores
Annelida	Polychaeta	Terebellida	Terebelliidae	*Amphitrite* sp.	Detritivores
Arthopoda	Malacostraca	Decapoda	Paguridae	*Pagurus acadianus*	Carnivorous
Arthropoda	Malacostraca	Decapoda		Unidentified shrimp crustose coralline-like	Carnivorous
Arthropoda	Malacostraca	Decapoda	Thoridae	*Lebbeus groenlandicus*	Carnivorous
Arthropoda	Malacostraca	Decapoda	Thoridae	*Lebbeus polaris*	Carnivorous
Arthropoda	Thecostraca	Balanomorpha	Balanidae	*Balanus balanus*	Active suspension feeder
Arthropoda	Thecostraca	Balanomorpha	Balanidae	*Semibalanus balanoides*	Active suspension feeder
Chordata	Ascidiacea	Aplousobranchia	Didemnidae	*Didemnum albidum*	Active suspension feeder
Chordata	Ascidiacea	Stolidobranchia	Styelidae	*Dendrodoa carnea*	Active suspension feeder
Cnidaria	Anthozoa	Actiniaria	Actiniidae	*Aulactinia stella*	Passive suspension feeder
Cnidaria	Anthozoa	Actiniaria	Actiniidae	*Cribrinopsis similis*	Carnivorous
Cnidaria	Anthozoa	Actiniaria	Actiniidae	*Urticina felina*	Carnivorous
Cnidaria	Anthozoa	Actiniaria	Actinostolidae	*Stomphia coccinea*	Carnivorous
Cnidaria	Anthozoa	Actiniaria	Hormathiidae	*Hormathia nodosa*	Carnivorous
Cnidaria	Anthozoa	Spirularia	Cerianthidae	*Pachycerianthus borealis*	Passive suspension feeder
Echinodermata	Asteroidea	Forcipulatida	Asteriidae	*Leptasterias cf. littoralis*	Carnivorous
Echinodermata	Asteroidea	Forcipulatida	Asteriidae	*Leptasterias polaris*	Carnivorous
Echinodermata	Asteroidea	Forcipulatida	Asteriidae	*Leptasterias* sp. (small)	Carnivorous
Echinodermata	Asteroidea	Forcipulatida	Asteriidae	*Stephanasterias albula*	Carnivorous

S1 Table continued. Megainvertebrates observed on quantitative transects in Nachvak Fjord during our surveys in 2022.

Phylum	Class	Order	Family	Taxa	Feeding guild
Echinodermata	Asteroidea	Spinulosida	Echinasteridae	*Henricia sanguinolenta*	Carnivorous
Echinodermata	Asteroidea	Spinulosida	Echinasteridae	*Henricia* sp.	Carnivorous
Echinodermata	Asteroidea	Valvatida	Solasteridae	*Crossaster papposus*	Carnivorous
Echinodermata	Asteroidea	Valvatida	Solasteridae	*Solaster endeca*	Carnivorous
Echinodermata	Echinoidea	Camarodonta	Strongylocentrotidae	*Strongylocentrotus droebachiensis*	Herbivorous/browser
Echinodermata	Echinoidea	Camarodonta	Strongylocentrotidae	*Strongylocentrotus pallidus*	Herbivorous/browser
Echinodermata	Holothuroidea	Dendrochirotida	Cucumariidae	*Cucumaria frondosa*	Passive suspension feeder
Echinodermata	Holothuroidea	Dendrochirotida	Cucumariidae	*Thyonidium drummondii*	Passive suspension feeder
Echinodermata	Holothuroidea	Dendrochirotida	Psolidae	*Psolus fabricii*	Passive suspension feeder
Echinodermata	Ophiuroidea	Ophiurida	Ophiophyrgidae	*Stegophiura nodosa*	Detritivores
Echinodermata	Ophiuroidea	Ophiurida	Ophiuridae	*Ophiura robusta*	Detritivores
Mollusca	Bivalvia	Adapedonta	Hiatellidae	*Hiatella arctica*	Active suspension feeder
Mollusca	Bivalvia	Myida	Myidae	*Mya truncata*	Active suspension feeder
Mollusca	Bivalvia	Pectinida	Pectinidae	*Chlamys islandica*	Active suspension feeder
Mollusca	Gastropoda		Lottiidae	*Testudinalia testudinalis*	Herbivorous/browser
Mollusca	Gastropoda	Neogastropoda	Buccinidae	*Buccinum scalariforme*	Carnivorous
Mollusca	Gastropoda	Neogastropoda	Buccinidae	*Buccinum undatum*	Carnivorous
Mollusca	Gastropoda	Neogastropoda	Muricidae	*Scabrotrophon fabricii*	Carnivorous
Mollusca	Gastropoda	Nudibranchia	Coryphellidae	*Coryphella verrucosa*	Carnivorous
Mollusca	Gastropoda	Trochida	Margaritidae	*Margarites helicinus*	Herbivorous/browser
Mollusca	Polyplacophora	Chitonida	Tonicellidae	*Tonicella marmorea*	Herbivorous/browser
Porifera				Unidentified blue Porifera	Active suspension feeder
