# Supplementary material for: Shallow subtidal marine benthic communities of Nachvak Fjord, Nunatsiavut, Labrador: A glimpse into species composition and drivers of their distribution
Source: PLoS One. 2023 Nov 9;18(11):e0293702. doi: 10.1371/journal.pone.0293702 (PMC10635441; doi:10.1371/journal.pone.0293702)
Supplement: S2 Table — Values are means with one standard deviation in parentheses. BOU = boulders, RwS = rocks with sediment, SwR = sediment with rocks, and UNC = unconsolidated, which included sand, gravel, or cobble. (PDF) [file pone.0293702.s002.pdf]

S2 Table. Numerical abundance (number m^{-2}) of megainvertebrates among habitats. Values are means with one standard deviation in parentheses. BOU = boulders, RwS = rocks with sediment, SwR = sediment with rocks, and UNC = unconsolidated, which included sand, gravel, or cobble.

Phylum	Taxa	BOU		RwS		SwR		UNC	
		Avg.	SD	Avg.	SD	Avg.	SD	Avg.	SD
Annelida	*Amphitrite* sp.	0.00	0.00	0.03	0.05	0.02	0.03	0.00	0.00
Annelida	*Golfingia (Golfingia) margaritacea*	0.00	0.00	0.01	0.01	0.00	0.00	0.00	0.00
Annelida	*Pectinaria gouldii*	0.00	0.00	0.00	0.00	0.18	0.06	2.00	0.00
Annelida	*Sabella* sp.	0.00	0.01	0.06	0.09	1.96	1.83	1.25	1.09
Arthopoda	*Pagurus acadianus*	0.00	0.00	0.01	0.03	0.06	0.06	0.00	0.00
Arthropoda	*Balanus balanus*	0.94	1.20	0.52	0.56	0.70	0.83	0.00	0.00
Arthropoda	*Lebbeus groenlandicus*	0.04	0.08	0.02	0.05	0.00	0.00	0.00	0.00
Arthropoda	*Lebbeus polaris*	0.23	0.09	0.12	0.09	0.08	0.05	0.00	0.00
Arthropoda	*Semibalanus balanoides*	0.08	0.13	0.82	1.70	1.46	0.83	0.00	0.00
Arthropoda	Unidentified shrimp crustose coralline-like	0.00	0.00	0.00	0.01	0.00	0.00	0.00	0.00
Chordata	*Dendrodoa carnea*	0.00	0.00	0.01	0.01	0.00	0.00	0.00	0.00
Chordata	*Didemnum albidum*	0.00	0.00	0.52	0.98	0.03	0.05	0.00	0.00
Cnidaria	*Aulactinia stella*	0.03	0.06	0.25	0.62	0.50	0.58	0.00	0.00
Cnidaria	*Cribrinopsis similis*	0.03	0.09	1.33	1.18	1.35	1.40	0.00	0.00
Cnidaria	*Hormathia nodosa*	0.00	0.00	0.26	0.47	0.00	0.00	0.00	0.00
Cnidaria	*Pachycerianthus borealis*	0.05	0.13	1.66	1.78	1.33	1.42	0.65	0.49
Cnidaria	*Stomphia coccinea*	0.00	0.00	0.11	0.31	0.00	0.00	0.00	0.00
Cnidaria	*Urticina felina*	0.00	0.01	0.02	0.03	0.03	0.03	0.00	0.00
Echinodermata	*Crossaster papposus*	0.00	0.00	0.01	0.01	0.04	0.03	0.00	0.00
Echinodermata	*Cucumaria frondosa*	0.00	0.00	0.00	0.01	0.00	0.00	0.00	0.00
Echinodermata	*Henricia sanguinolenta*	0.00	0.00	0.00	0.01	0.00	0.00	0.00	0.00
Echinodermata	*Henricia* sp.	0.00	0.00	0.02	0.03	0.00	0.00	0.00	0.00
Echinodermata	*Leptasterias cf. littoralis*	0.00	0.01	0.04	0.05	0.02	0.03	0.10	0.03
Echinodermata	*Leptasterias polaris*	0.01	0.01	0.07	0.06	0.07	0.06	0.07	0.01
Echinodermata	*Leptasterias* sp. (small)	0.00	0.00	0.04	0.10	0.00	0.00	0.00	0.00

S2 Table continued. Numerical abundance (number m^{-2}) of megainvertebrates among habitats.

Echinodermata	*Ophiura robusta*	0.00	0.00	0.00	0.00	0.07	0.05	0.01	0.01
Echinodermata	*Psolus fabricii*	0.13	0.30	0.28	0.46	0.01	0.01	0.00	0.00
Echinodermata	*Solaster endeca*	0.00	0.00	0.00	0.00	0.01	0.01	0.01	0.01
Echinodermata	*Stegophiura nodosa*	0.00	0.00	0.00	0.00	0.01	0.01	0.00	0.00
Echinodermata	*Stephanasterias albula*	0.00	0.00	0.04	0.07	0.00	0.00	0.00	0.00
Echinodermata	*Strongylocentrotus droebachiensis*	3.24	5.65	30.16	9.20	28.30	8.70	24.00	1.13
Echinodermata	*Strongylocentrotus pallidus*	0.00	0.01	0.05	0.06	0.00	0.00	0.00	0.00
Echinodermata	*Thyonidium drummondii*	0.00	0.00	0.00	0.01	0.00	0.00	0.00	0.00
Mollusca	*Buccinum scalariforme*	0.00	0.00	0.03	0.07	0.04	0.05	0.00	0.00
Mollusca	*Buccinum undatum*	0.00	0.00	0.09	0.14	0.01	0.01	0.00	0.00
Mollusca	*Chlamys islandica*	0.00	0.00	0.02	0.03	0.00	0.00	0.00	0.00
Mollusca	*Coryphella verrucosa*	0.00	0.00	0.00	0.00	0.00	0.00	0.03	0.04
Mollusca	*Hiatella arctica*	0.00	0.00	0.02	0.05	0.00	0.00	0.00	0.00
Mollusca	*Margarites helicinus*	49.11	48.86	0.03	0.06	0.00	0.00	0.00	0.00
Mollusca	*Mya truncata*	0.00	0.00	0.90	1.78	1.70	1.90	0.00	0.00
Mollusca	*Scabrotrophon fabricii*	0.00	0.00	0.00	0.00	0.01	0.01	0.00	0.00
Mollusca	*Testudinalia testudinalis*	0.05	0.03	0.41	0.59	0.26	0.26	0.23	0.18
Mollusca	*Tonicella marmorea*	0.00	0.00	0.25	0.23	0.03	0.05	0.00	0.00
Porifera	Unidentified blue Porifera	0.00	0.00	0.00	0.01	0.00	0.00	0.00	0.00
